# Magnesium and Vascular Changes in Hypertension

**DOI:** 10.1155/2012/754250

**Published:** 2012-02-29

**Authors:** Ana Rosa Cunha, Bianca Umbelino, Margarida L. Correia, Mario Fritsch Neves

**Affiliations:** Department of Clinical Medicine, University Hospital Pedro Ernesto, State University of Rio de Janeiro, Avenida 28 de Setembro, 77 Sala 329, 20551-030, Rio de Janeiro, RJ, Brazil

## Abstract

Many factors have been implicated in the pathogenesis of hypertension, including changes in intracellular concentrations of calcium, sodium, potassium, and magnesium. There is a significant inverse correlation between serum magnesium and incidence of cardiovascular diseases. Magnesium is a mineral with important functions in the body such as antiarrhythmic effect, actions in vascular tone, contractility, glucose metabolism, and insulin homeostasis. In addition, lower concentrations of magnesium are associated with oxidative stress, proinflammatory state, endothelial dysfunction, platelet aggregation, insulin resistance, and hyperglycemia. The conflicting results of studies evaluating the effects of magnesium supplements on blood pressure and other cardiovascular outcomes indicate that the action of magnesium in the vascular system is present but not yet established. Therefore, this mineral supplementation is not indicated as part of antihypertensive treatment, and further studies are needed to better clarify the role of magnesium in the prevention and treatment of cardiovascular diseases.

## 1. Introduction

Primary hypertension is the most common form of blood pressure elevation whose cause remains unknown. However, many factors have been implicated in its pathogenesis, such as the renin-angiotensin-aldosterone system and the sympathetic nervous system hyperactivation. In addition, changes in intracellular ions such as calcium, sodium, potassium, and magnesium have also been related to high blood pressure.

In the last years, the prevalence of hypertension is around 25–30% in developed countries [1], and several treatments have been proposed for the BP control and prevention of its onset. Among the various studies concerning non-pharmacological treatments, there is need for lifestyle change with the inclusion of regular physical activity and healthy eating habits.

Observational studies have shown that a diet rich in potassium, magnesium, and calcium, present mainly in fruits and vegetables, is associated with lower incidence and mortality from cardiovascular disease [2]. In particular, magnesium has been the target of many studies [3], considering that there is a significant inverse correlation between serum magnesium levels and incidence of cardiovascular diseases [4]. In addition, hypertensive patients generally exhibit reduced intracellular concentrations of magnesium, while the contents of sodium and calcium are often increased compared to normotensive subjects [5, 6].

The dietary recommendation (Recommended Dietary Allowances/RDA) for magnesium is 400 to 420 mg daily for adult men and 310 to 320 mg daily for adult women. However, consumption is far below this recommendation, and the high prevalence of this deficiency has been associated to several chronic diseases. Magnesium is found in most foods, but in varying concentrations. Leafy vegetables, nuts, whole grains, fruits, and legumes are considered as foods with high-magnesium concentrations [7].

In order to gather more information about the association of magnesium with cardiovascular diseases, we performed a narrative review of the literature through the PubMed database with the following descriptor: magnesium, intracellular magnesium, hypertension, arterial stiffness, and endothelial function. We included narrative reviews, experimental protocols, and controlled studies in the last 15 years (1996–2011), and case reports were excluded.

## 2. Physiological Functions and Pathophysiological Actions of Magnesium

**Figure 1:**
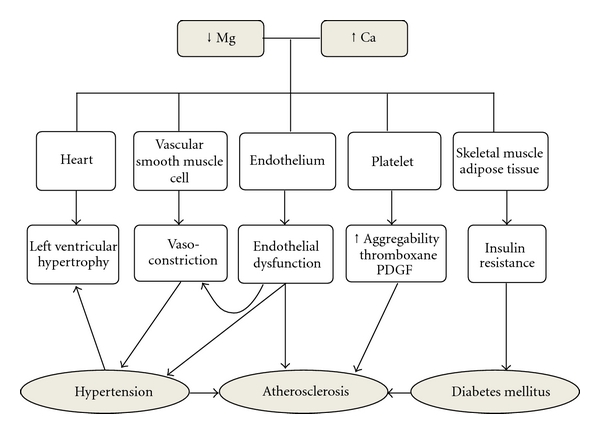
Role of magnesium and calcium in the pathophysiology of hypertension, diabetes mellitus, and atherosclerosis.

The mineral magnesium is the second most abundant intracellular cation and is involved in several important biochemical reactions [8]. It is known that magnesium has antiarrhythmic effect and can influence blood pressure levels by modulating vascular tone. Changes in extracellular magnesium content are able to modify the production and release of nitric oxide (NO), resulting in the alteration of arterial smooth muscle tone by affecting calcium concentrations. Magnesium also participates in glucose metabolism and insulin homeostasis. For these reasons, it has been suggested that magnesium deficiency or changes in its metabolism are related to the pathophysiology of hypertension, atherosclerosis, insulin resistance, and diabetes (Figure 1) [9].

Increased levels of extracellular magnesium inhibit calcium influx. Conversely, reduced extracellular magnesium activates calcium influx via calcium channels. Low intracellular magnesium concentrations stimulate inositol-trisphosphate-(IP3-) mediated mobilization of intracellular calcium and reduce Ca^2+^-ATPase activity. Thus, calcium efflux and sarcoplasmic reticular calcium reuptake are reduced, leading to cytosolic accumulation of calcium and increased intracellular calcium concentration, which is a crucial factor for vasoconstriction. Increased intracellular levels of magnesium result in decreased intracellular free calcium concentration promoting vasodilation [10]. The action of magnesium as a calcium channel blocker may also help to reduce the release of calcium and thus reducing vascular resistance. In addition, magnesium also activates the Na-K ATPase pump that controls the balance of these minerals contributing to the homeostasis of electrolytes in cells [11].

Smaller concentrations of magnesium seem to be associated with reduced serum HDL-cholesterol along with increased LDL-cholesterol and triglycerides levels [9]. Additionally, deficiency of this mineral has been previously related to oxidative stress, proinflammatory state, endothelial dysfunction, platelet aggregation, insulin resistance, and hyperglycemia [12].

High levels of magnesium may increase production of adenosine triphosphate (ATP) and intracellular glucose utilization, since magnesium acts as a cofactor of all reactions involving ATP transfer [13]. Insulin seems to be one of the most important factors that regulate plasma and intracellular magnesium concentrations. It has been suggested that an ATPase-dependent pump is involved in the mechanism by which insulin regulates the erythrocyte magnesium content [14]. On the other hand, intracellular magnesium may play a role in modulating insulin-mediated glucose uptake and vascular tone. Reduced urinary magnesium losses have been implicated in better metabolic control [15]. Low plasma and intracellular magnesium levels may contribute to reducing insulin sensitivity. In fact, suppression of intracellular free magnesium concentrations is known to decrease cellular glucose utilization and thus to promote peripheral insulin resistance as a postreceptor defect [16].

Concerning insulin homeostasis, there is a hypothesis that there is increased secretion of insulin and adrenaline in hypomagnesemia in order to maintain magnesium and cellular cAMP (3′,5′-cyclic adenosine monophosphate) concentration [17]. Furthermore, the intracellular concentration of magnesium appears to be dependent on the extracellular level, and its influx through calcium channel is voltage dependent. Extracellular magnesium can competitively inhibit calcium channels and determine reduced secretion of insulin. This inhibition does not occur when there is no magnesium in the extracellular space, resulting in higher insulin secretion [18].

Some studies suggest the possible role of intracellular magnesium on the activity as a regulator of the main communication channels of the cell membrane, suggesting that there may be an association between changes in intracellular content of ions induced by supplementation of magnesium and its antihypertensive effects [19].

## 3. Magnesium and Blood Pressure

Experimental models of hypertension have been associated with reduced serum and tissue levels of magnesium. In spontaneously hypertensive rats (SHRs), increase of blood pressure arises from the age of young adults, around 12 to 16 weeks of life, being attributed to a genetic component similar to human essential hypertension [20]. In SHR, and also in DOCA-salt model, reduced levels of intracellular magnesium have been noted in smooth muscle cells and cardiomyocytes.

Magnesium supplementation had little antihypertensive effect in adult SHR with well-established hypertension. In fact, the effect of supplementation was only positive in younger animals, when started in the prehypertensive phase, preventing or at least attenuating the development of hypertension [21]. This finding is highly suggestive of a more protective effect of supplemental magnesium, which may prevent or slow the rise in blood pressure at an early stage of hypertension.

In other experimental studies, dietary magnesium deficiency was associated with increased blood pressure in previous normotensive animals, and magnesium supplementation was able to reverse this condition. However, clinical trials of magnesium supplementation in hypertensive patients show divergent results. Some studies demonstrate low serum magnesium levels in hypertensive patients when compared with normotensive subjects, and blood pressure levels reduction after magnesium supplementation [3], although other studies have not confirmed this finding. For this reason, while adequate intake of magnesium through diet is recommended, supplementation of this mineral is not indicated as part of antihypertensive treatment [22, 23]. 

Experimental, clinical, and epidemiological studies have observed a close inverse relationship between dietary intake or supplementation of magnesium and blood pressure level, indicating the potential role of magnesium deficiency in the pathogenesis of essential hypertension [24], but the mechanism is unclear. The effects of magnesium on the smooth muscle cells growth and inflammation may be important.

A relationship has also been reported between the rennin-angiotensin system, magnesium, and blood pressure. Hypertensive patients with high renin activity have significantly lower serum magnesium levels than normotensive subjects, and plasma renin activity is inversely associated with serum magnesium [25]. Hypertensive patients without blood pressure control may have hypomagnesemia. Hatzistavri and colleagues have shown that magnesium supplementation was associated with slight reduction of 24 h blood pressure levels in patients with mild hypertension [3], which can be evaluated by ambulatory blood pressure monitoring [26]. On the other hand, a study comparing the relationship between serum magnesium, vascular dysfunction, hypertension, and atherosclerosis has not shown enough results to support this association, indicating that low serum magnesium cannot be considered a risk factor for development of these conditions [27]. 

## 4. Magnesium and Vascular Structure

Hypertension is also associated with unfavorable changes in elastic properties of large arteries. Some studies have shown the independent prognostic role of arterial stiffness in cardiovascular events in hypertensive patients, which can be assessed by measurements of the pulse wave velocity (PWV) [33–35]. However, there are a few studies showing the influence of magnesium in this condition so far. Van Laecke and colleagues have reported that serum hypomagnesemia associated with hypertension, endothelial dysfunction, dyslipidemia, and inflammation may affect vascular stiffness in patients who underwent kidney transplantation since the low serum magnesium was independently associated with PWV assessed by SphygmoCor [36]. In an experimental study evaluating the structure of the carotid artery in rats, magnesium deficiency was associated with hypertrophic vascular remodeling, which was attenuated by supplementation of this ion. These findings suggest that magnesium deficiency alters the vascular mechanical properties in young animals and may be a mechanism involved in the pathogenesis of hypertension, atherosclerosis, and other cardiovascular diseases [37].

Other possible mechanisms of magnesium action are anti-inflammation, antioxidion, and modulation of cell growth properties. In fact, the production of reactive oxygen species is usually increased in the vasculature of hypertensive patients, and the involvement of magnesium could occur through the reduction of inflammation and oxidative stress [38]. Magnesium has antioxidant properties that could attenuate detrimental effects of oxidative stress on the vasculature, thereby preventing increased vascular tone and contractility [39].

## 5. Magnesium and Vascular Function

Endothelial dysfunction refers to an imbalance in the endothelial production of mediators that regulate vascular tone, platelet aggregation, coagulation, and fibrinolysis. There is a worsening in the endothelium-dependent relaxation, which can be caused by both loss of NO bioavailability as changes in the production of other endothelium-derived vasoactive substances mainly endothelin-1 and angiotensin II.

The role of magnesium in the endothelial dysfunction has been discussed elsewhere. Indeed, it has been reported that magnesium modifies the vascular tone by regulating endothelium and smooth muscle cell functions along with an important role in the classical pathway of NO release. Experiments in animals have also showed increased production of prostacyclin and NO by magnesium, promoting endothelium-independent and endothelium-dependent vasodilation [40].

The peripheral vascular resistance may be modified by magnesium, also through the regulation of responses to vasoactive agents, particularly angiotensin II, endothelin, and prostacyclin. Animals deficient in magnesium have presented high levels of endothelin-1, whose values have been reduced after supplementation of this mineral [41].

A study that followed more than 90,000 postmenopausal women showed that dietary magnesium intake was inversely associated with plasma concentrations of inflammatory markers such as interleukin-6, C-reactive protein (CRP), and tumor necrosis factor-*α* [7]. This same study emphasized that magnesium intake could improve endothelial dysfunction and inflammation and might play a role in preventing metabolic syndrome.

There are a few studies demonstrating the relationship between magnesium supplementation, endothelial function, arterial stiffness, and carotid intima-media thickness. Some reports point out beneficial effects of magnesium supplementation in improving endothelial function in the brachial artery in patients with coronary artery disease [42], heart failure [43], and diabetes mellitus [44], while others show favorable outcome of magnesium supplementation through improvement of insulin sensitivity [45, 46].

## 6. Magnesium Supplementation

Magnesium can be supplemented in different ways, such as oxide, hydroxide, chelate, sulfate, and citrate. Magnesium sulfate, for example, can be used as anticonvulsant therapy in preeclampsia due to its neuroprotective action and a possible role in regulating vascular tone [47].

Some studies have shown blood pressure lowering after magnesium supplementation. The administration of magnesium oxide (400 mg daily) for eight weeks in patients with hypertension can reduce blood pressure levels, and this reduction has already been detected in office measurements and by ambulatory blood pressure monitoring [29]. A study of 48 subjects has demonstrated that 600 mg of magnesium pidolate per day was able to reduce blood pressure levels in the supplemented patients when compared to the group with no supplementation [3]. This same dosage of supplement was also associated with reduction of serum total cholesterol, LDL-cholesterol, and triglycerides and improvement of insulin resistance.

Haenni and colleagues reported positive effects of magnesium supplementation in order to confirm the relationship between the metabolism of this mineral and alteration of endothelial function by showing increased endothelium-dependent vasodilatation after magnesium infusion [48]. Furthermore, another study showed that chronic magnesium supplementation was able to improve endothelial function in patients with coronary artery disease [42]. Some positive and negative results after magnesium supplementation are shown in Table 1. A meta-analysis evidenced a weak causal correlation between magnesium supplementation and blood pressure reduction, and double-blind placebo controlled trials are needed to determine the effect of magnesium supplementation on cardiovascular outcomes [49].

## 7. Conclusions

Magnesium is a mineral with important functions in the body, and it is important that their levels are adequate. The conflicting results of studies evaluating the effects of magnesium supplements on blood pressure and other cardiovascular outcomes indicate that the action of magnesium in the vascular system is present but not yet established. Certainly, the lack of definitive conclusions due to heterogeneity of study populations with different clinical profiles and severity of illness, lack of standardization of the type of supplement and the dose, and, finally, very short time of treatment, most often between one and three months, are factors that contribute to the difficulty to achieve the primary objectives. Based on recent studies, although we cannot make categorical statements, it appears that magnesium is more involved in the functional vascular changes, and also on local metabolic stability with no influence on the vascular structure. Therefore, further studies are needed to evaluate the risk of magnesium deficiency and the effects to be considered in this mineral supplementation.

Possibly the most important point is to define a more homogeneous study population, considering the same gender and age range, dosage, and type of supplement, as well as a longer period for supplementation. After resolving these concerns, it will be possible to clarify the role of magnesium in the prevention and treatment of cardiovascular diseases.

## Figures and Tables

**Table 1:** Positive and negative results with magnesium supplementation for blood pressure (BP) reduction.

Study population	Mg supplementation	Comparator group	Duration of treatment	Clinical outcome	Year [Reference]
24 patients with uncomplicated hypertension	600 mg of magnesium pidolate	24 age- and sex-matched controls	12 weeks	Small but significant reductions in mean 24 h systolic and diastolic BP levels	2009 [18]
35 patients with essential hypertension	magnesium 70.8 mg/d; potassium 217.2 mg/d	32 patients received lacidipine (4 mg/d)	4 weeks	Systolic and diastolic BP decreased, and small arterial compliance values increased	2006 [28]
60 hypertensive patients	20 mmol/d magnesium oxide	60 hypertensive patients in a control period, crossover design	8 weeks	Office, home, and average 24-hour BPs were significantly lower in the magnesium supplementation period	1998 [29]
15 patients with mild to moderate primary hypertension	600 mg/day of oxide magnesium	15 hypertensive patients in a crossover design, receiving placebo	6 weeks	Significant reduction of systolic, diastolic, and mean BP	1996 [19]
698 healthy adults with high-normal diastolic blood pressure	360 mg of magnesium (diglycine)	1 g of calcium (carbonate)	6 months	Neither calcium nor magnesium produced significant changes in BP at 3 and 6 months	1995 [30]
14 mild to moderate hypertensives	Magnesium pidolate (15 mmol/day)	Placebo	6 months	Magnesium supplementation does not affect BP at rest and during sympathetic stimulation	1992 [31]
71 subjects with mild hypertension or a high-normal BP	15 mmol Mg	Placebo	6 months	No general effects on the BP	1991 [32]
